# The Sacred Power of Beauty: Examining the Perceptual Effect of Buddhist Symbols on Happiness and Life Satisfaction in China

**DOI:** 10.3390/ijerph17072551

**Published:** 2020-04-08

**Authors:** Zhenzhen Qin, Yao Song

**Affiliations:** 1 School of Journalism and Communication, Anhui Normal University, Wuhu 241002, China; qinzhenzhenad@ahnu.edu.cn; 2 School of Design, The Hong Kong Polytechnic University, Hong Kong 00852, China

**Keywords:** well-being, Buddhism, life satisfaction, happiness, religiosity, symbol design

## Abstract

The theoretical relationship between Buddhism and subjective well-being has gained much academic attention in recent decades. However, the prominent determinants of religiosity remain limited for researchers to understand a holistic picture of religion-informed subjective well-being, particularly in the context of Buddhism. This study has applied a quantitative survey to verify the impact of the aesthetic effects of the Buddhist gesture symbol on Chinese people’s subjective happiness through sequential mediators of life satisfaction and the perceived religiosity. The significance of this study is threefold. Firstly, it aims to enrich the current academic understanding of the religion-informed subjective well-being by introducing a new determinant of the Buddhist symbols. Secondly, the current study investigates the mechanism of how Buddhist symbols could influence happiness by analyzing the sequential mediating roles of religiosity and life satisfaction. Thirdly, this study empirically examines the topic in the context of China to confirm and underpin the theoretical relationship between Buddhism and subjective well-being in relevant research, which has previously focused on Western culture. Our results indicated that the aesthetic perception of the Buddhist gesture symbol positively influenced perceived happiness and life satisfaction. In addition, perceived religiosity and life satisfaction sequentially mediated the perceived happiness after seeing the Buddhist gesture symbol. Our findings contribute to the current academic understanding of religious symbols and their impacts on subjective well-being.

## 1. Introduction

The theoretical relationship between religion and human mental health has gained much academic attention in recent decades [1,2]. The role that religion plays in the understanding of and reacting to lives has been studied in psychology [3], social behavior [4], cultural studies [5,6], and so forth. Scholars argue that religious affiliations can positively predict the indicators of subjective well-being, such as happiness [7] and life satisfaction [8], to maintain human mental health [9]. Among these, Buddhism has been considered as a distinctive resource for people to interpret and respond to life suffering [10]. Much research has integrated Buddhist approaches into psychological health treatments because of its power of spiritual techniques (e.g., mindfulness meditation), which differs Buddhism significantly from Western rational positivism [11,12,13,14,15,16,17]. Buddhist practices, over the past 2500 years, have been entangled with cultivating mental well-being through identifying the inner causes of human suffering and the means of freedom from suffering [18]. 

The visually aesthetic effect, as a fundamental discourse in religion [19,20,21], is of importance to foster positive attitudes. The aesthetic effect of Buddhist artworks has worked as a prevalent way to teach people Buddhist doctrines in history and construct its profound meaning system through figurative quality [19,20]. Buddhist symbols in artworks, particularly the symbol of the Buddha gesture, can embody and symbolize the intangible meanings and powers rooted in Buddhist spirituality with beautiful decoration [21,22,23]. Empirical research [24] suggests that religious symbols impact positive health behaviors; however, the aesthetic effect is underestimated with limited evidence, especially in the case of Buddhist symbols. As a sensible medium, Buddhist symbols tend to encourage people’s conceptual processing of their religiosity through enriched comprehension. Though the aesthetic power of Buddhist gesture symbols has been previously discussed [22,23,24,25,26,27], limited empirical evidence has been provided to underpin the theoretical relationship between the aesthetics of Buddhist symbols and relevant psychological constructs and processes.

Furthermore, scholars have construed that perceived religiosity tends to predict people’s perceptual subjective well-being since religious belief and practice can influence people’s lives through shaping their beliefs, knowledge, and attitudes [28]. However, studies on the relationship between expressions of Buddhism and dimensions of religiosity (e.g., practices and attitudes) have been limited. Previous measurements of religiosity have been widely applied in Western cultures to examine people’s attitudinal perceptions of religion [14], such as Christianity [29] and Islam [30]. Moreover, some recent studies report an insignificant relationship between people’s subjective well-being and religious factors (e.g., religiosity). The term subjective well-being describes and measures affective and cognitive satisfaction components of a person’s life [31]. Francis et al. [32] reported an insignificant relationship between Christian religiosity and happiness among German students, contrary to the evidence of the positive correlation between happiness and Christian religiosity [33]. Considering the rare previous investigations of effects of Buddhist religiosity on subjective well-being and contrary observations in the context of Christianity, it is necessary to examine whether Buddhist religiosity plays an effective role in influencing constructs of subjective well-being.

China, with the potential largest Buddhist population globally, has seldom been examined empirically in the research of Buddhism. According to a recent report [34], there are around 102,000,000 Buddhists in China as of 2019. Buddhism arrived in China shortly before the Christian era [35]. For over 1900 years, Buddhism has become part of Chinese culture at various levels ranging from philosophy to arts. For this reason, the Chinese are quite familiar with Buddhism and regard it as one part of their daily lives [36]. Symbolic figures and images from Buddhism are prevalent in artistic works [37] and daily decorations [38,39] to evoke the cognitive effect of meaning-making. It has been suggested that the color symbolism in China is influenced by Buddhist traditions [40]. Though a positive relationship between religiosity and subjective well-being is reported, some recent scholars [41] reported a negative relationship between religion (i.e., religious participation of Buddhism, Taoism, Islam, Catholicism, and Protestantism) and subjective well-being among the elderly in China. An empirical study, to address these previous nuanced results about Buddhist religiosity and subjective well-being in the Chinese population, is critical to understand the interactive relationship between Buddhist factors and subjective well-being. 

Thus, this study applied a quantitative survey to verify the impact of Buddhist aesthetic symbols on Chinese people’s perceived religiosity and subjective well-being. The significance of this study is threefold. Firstly, it aims to enrich the current academic understanding of the religion-informed subjective well-being by introducing the new determinant of Buddhist aesthetic symbols. Secondly, the current study investigates the mechanism that Buddhist symbols could influence subjective well-being by separating the subjective well-being and analyzing the sequential mediating role of religiosity and life satisfaction. Thirdly, this study empirically examines the topic in the context of China to confirm and underpin its theoretical relationship in relevant research, which has previously focused on Western culture. 

## 2. Literature Review

In this section, the theoretical background of the present study will be discussed. Firstly, we introduce the definitions and concepts of the Buddhist symbol and how the aesthetic perceptions of Buddhist symbols can improve people’s attitudes. Secondly, the perceived religiosity is introduced to propose its hypothetical relationship to the Buddhist symbol. The current research on religiosity and subjective well-being will be discussed thoroughly. Finally, from the perspective of subjective well-being, we argue that the Buddhist aesthetic symbols are interlinked with people’s happiness through the mediators of Buddhist religiosity and life satisfaction.

### 2.1. Buddhist Gesture Symbols and Aesthetics

The theory of semiology has been studied as a fundamentally non-verbal tool and approach to communicating human knowledge [42,43,44]. Symbols, as one main category of the symbolic sign system, can signify a specific meaning shaped in society [45]. An individual can extract and process image schemas as perceptual symbols to construct conceptual interpretation [46,47]. Recent studies have shed light on the unique functions of symbolic signs on users’ semantic comprehension in the field of art and design [48,49,50]. The creations of symbolic representation have been suggested as a process of transforming any intangible concepts into perceivable forms [51]. 

A religious symbol has been conceived and designed to represent essentially conceptual characteristics of a specific religion in history through its figurative quality [52]. It facilitates an individual to comprehend and perceive meaningful spirituality [53]. Recent empirical studies in social science provide preliminary evidence that religious symbols work as an effective factor to foster positive attitudes [54,55,56,57,58]. For example, the symbol of the goddess Durga in Hinduism has worked as a visual cue to increase the reporting intention of violence [59]. The aesthetic perception is proposed, though with scarce empirical evidence, as a fundamental factor to bridge the theoretical relationship between religious symbols and positive attitudes [19,20,21]. Previous scholars [60] construe that a religious belief is an embodied form of supreme beauty. Consistent with this perspective, Buddhist symbols work as a sensible medium communicating the cognitive response of aesthetic pleasure attached with Buddhist meanings [61]. 

Buddhist symbolism is of importance to construct the profound meaning system of Buddhism [62,63,64]. For this reason, Buddhist symbols have been utilized and manifested in many Buddhist artworks and sites to propagate the divine power and presence of Buddha [65]. It has been suggested that a Buddhist symbol works as an effective medium to map the spiritual world into reality through the aesthetic figurative quality [53,66,67,68]. Though the symbols in Buddhism are diverse across cultures, the Buddha gestures originate from Mudra in Indian Buddhism, and have been acknowledged to play a central role in embodying the essences of original Buddhist philosophy [69]. The gesture symbols have been used, particularly in iconography, to evoke a specific episode deriving from Buddhist legends (e.g., Nianhua Weixiao in Chinese) and to establish divinities [24,64]. A specific symbol of gesture in Buddhism is originally associated with ritualistic meanings from Buddhist ceremonies. Each of the fingers is attached with specific meaning and energy in Buddhism [70]. It has been believed by Buddhists that there are some supernatural powers to transcend joy and pleasure when a particular gesture is made [71]. For years, its iconographic meanings have evolved and come down through various artistic representations in China [72,73]. For instance, one of the frequently figured gestures found in Mogao Cave at Dunhuang China (e.g., Cave No.435 and Cave No.321) is the left hand raised holding a flower using the middle and ring fingers, called Nianhua Shou in Chinese [19]. This gesture has symbolized the enlightenment when thoroughly comprehending the profound law in Buddhism, which originates from the Buddhist legend of Nianhua Weixiao in China. It describes that dharma presented a flower without saying a word. All disciples were confused about his intention; only Kassapa smiled and indicated that he was the only one grasping the underlying meaning from dharma [74]. Later, this gesture symbol has been utilized to signify a subtle state of contentment and calmness as a result of enlightenment. In addition, the flower in this gesture has been gradually rendered as a lotus to strengthen the iconographic meanings, since the symbol of the lotus has represented the purity through the meditation process [75,76]. 

Recently, the aesthetic power of the Buddhist gesture symbol has been emphasized in the related literature [22,23,24,25,26,27,72]. Scholars, like Deng [38], have examined how the Buddhist gesture symbol has been utilized and adapted to enhance people’s aesthetic satisfaction and life quality through psychological enjoyment and pleasure elicited by Buddhist spirituality. For example, it has been presented on design objects aiming to significantly influence people’s attitudinal behaviors. Nevertheless, the empirical effects of Buddhist symbols, particularly the gesture symbol, seldom have been examined. Therefore, it is difficult for researchers to identify its significance and evidence to nourish further studies. Moreover, religiosity, as a critical measurement tool to examine the relationship between religion, happiness, and life satisfaction [1,28], is rarely involved in the topic of aesthetic effects of Buddhist factors. 

### 2.2. Perceived Religiosity in the Buddhist Context

The notion of religiosity has been widely adopted by Western scholars in theology [14], psychology [77], and sociology [78] to examine people’s perceptions of religion. The definition of religiosity refers to the extent to which an individual is devoted to religion [29]. Many scholars have discovered the distinctive effects of religiosity of Western religions on an individual’s attitudes and behaviors. For instance, previous literature has shown that individual perception of religiosity plays a significant role in attitudes towards life [79]. Schwartz and Huysmans [80] noted that religiosity is prominently related to people’s value priorities. Collective behavior is associated with people’s religiosity [81]. Previous studies mainly focus on the influence caused by perceived religiosity; however, the determinants predicting perceived religiosity remain ambiguous for researchers to understand the mechanism of how religions influence people’s life. 

Since the majority of studies on religiosity has been conducted in Western religions, such as Christianity [29], research into Buddhist spirituality is limited in exploring the degree to which people are committed to Buddhism [82]. Recently, scholars have started to adopt the factor of religiosity in the context of Buddhism in several academic disciplines [83]. Buddhist teachings, such as life-suffering and self-adjustment, promote the spiritual tolerance to increase the resilience to live [10,84]. For instance, Buddhist religiosity has been increasingly considered in psychological health treatments because Buddhist teaching of life suffering can particularly transform people’s life attitude in the trauma recovery process [11,85]. Nevertheless, the theoretical relationship between Buddhist religiosity and life resilience has remained unclear. An empirical confirmation in the context of Buddhism, which is significantly different from Western rational positivism [11], contributes to helping researchers gain a holistic picture of the effect of religiosity in different religious contexts.

Although some scholars have provided evidence to understand the determinants of Buddhist religiosity, current studies on these remain ambiguous. For example, Yeung and Chow [86] have qualitatively examined the sources, recourse, and positive consequences of Buddhist religiosity. The physical medium, like electronic print media, has been found as a principal source to construct the Buddhist religiosity in their study. However, it lacks a deeper understanding of the factors produced by those media. Some scholars have suggested that psychological changes can improve Buddhist religiosity [87]. Mullen and Rinpoche [88] discussed how compassionate imagery used in Buddhism influences perceptions. This leaves the research question of whether aesthetical perceptions evoked by Buddhist imagery would influence people’s Buddhist religiosity. 

As aforementioned in Section 2.1, Buddhist symbols would improve peoples’ aesthetic pleasure. Relevant observations have been made in the research on Christianity; for instance, Meyer [89] argues that the sensational appeal of churches can encourage the aesthetics of persuasion in Christianity. He has construed that aesthetic forms are associated with the belonging of religions. Consistent with this perspective, Lazarus [15] noted that the spiritual interaction between the principal figures and believers is emphasized in the traditional African spiritual system. Given this, it is hoped to examine the Buddhist aesthetic symbols, as a potential determinant, to improve perceived religiosity of Buddhism. In addition, addressing the question of whether increased religiosity predicts health outcomes contributes to the current literature related to the role of religion in improving well-being. 

### 2.3. Buddhism, Subjective Well-being, Life Satisfaction, and Happiness 

The theoretical relationship between religions and subjective well-being can be traced to Durkheim’s seminal research on how religious involvement can influence the rates of suicide [90]. The concept of subjective well-being mainly concerns with people’s attitudinal perceptions of their lives in positive ways [31,91,92,93]. Studies on happiness and life satisfaction have been conducted to enhance people’s mental health as a universal goal [94,95], which means that individual health status is not merely the absence of physical body quality, such as illness. The social function of religions, in this context, is highly relevant to improve the mental status by a positive attitude toward life. Consequently, the determinants of subjective well-being rooted in religious beliefs require greater scientific attention to build the theoretical connection to underpin religion-informed subjective well-being. 

Previous literature has suggested that various aspects of well-being tend to be improved by religions because the role of religions has been regarded as a happy-orientated experience [96]. The current theoretical foundation on well-being has been derived from two threads: the hedonic approach, which focuses on happiness through pleasure attainment and pain avoidance; and the eudaimonic approach, which focuses on meaning and self-realization, and defines well-being in terms of the degree to which a person is fully functioning [97]. Given this, scholars [98] construe that religious factors work as a spiritual approach to enhance hedonic and eudaimonic well-being. For instance, Ellison [8] has examined the mediating role of religion in easing stress and improving well-being, which means that people with a stronger faith in religions can overcome traumatic events easier. Moreover, the Oxford Happiness Inventory has shown that religiosity tends to work as a crucial mediator to enhance people’s perceptual subjective well-being. The reason lies in the critical role of religious commitment in people’s lives through shaping their beliefs, knowledge, and attitudes [28]. People’s religious commitments and beliefs influence feelings and attitudes towards life [99]. It motivates people to do things for one’s “True Self”, which is consistent with the view of eudaimonic well-being [98]. However, recent literature suggests contradictory results on the relationship between subjective well-being and religion [100]. Some studies report an insignificant relationship between happiness and religious effects [32,101]. This encourages further study to confirm the relationship in the current context of Buddhism. 

As aforementioned in Section 2.2, Buddhist thoughts have highly emphasized spiritual adaptation and self-adjustment against life sufferings [10]. Buddhist practice, such as mindfulness meditation, aims to calm the mind and observe the various components of unsatisfactory consciousness more objectively, and with increasing spiritual ability aimed toward the cessation of suffering [102]. This cognitive attitude formation process is linked to a happy-oriented life. For instance, the Dalai Lama [103] construes that life attitudes suggested by Buddhism are to overcome inevitable adversities and sufferings to achieve greater happiness. Other scholars argue similarly that Buddhism is a happiness-oriented faith in the perspective of emotional self-adjustment, which is consistent with eudaimonic well-being in terms of self-realization [18]. Richard [104] suggests that Buddhism stresses a cognitive process of enhanced awareness of negative emotions, understanding that it is but a thought, then processing it by inner transformation into positive mental status. In this view, Buddhist religiosity is the potential to actively build attitudinal reactions to life suffering and negative emotions towards perceptual subjective well-being in terms of happiness and life satisfaction.

Religious symbols, religiosity, and subjective well-being are interwoven. Durkheim [13] underlines the effects of religious symbols—through transferring the collective energy of religiosity onto a visible object—on uniting and enlightening people who have shared beliefs. Given this, considering the aesthetic effects of Buddhist gesture symbols on enjoyment and pleasure elicited by Buddhist spirituality [22,23,24,25,26,27,72], we hypothesize that Buddhist gesture symbols facilitate people’s religiosity. In addition, the mediator of religiosity has been previously studied to impact subjective well-being. For example, Barton et al. [105] reported that adolescent religiosity mediates the relationship between parental religiosity and adolescent well-being in the context of Christianity. Noteworthy of mention is that women showed a higher level of religiosity than men [106]. Given this, Buddhist religiosity is firstly hypothesized to mediate the relationship between the aesthetic perception of Buddhist gesture symbols and subjective well-being. We argue that the visual expression of Buddhist symbols influences religiosity and then enhances subjective well-being. 

Furthermore, the internal mechanism of subjective well-being remains ambiguous in current research; to be more specific, the relationship between life satisfaction and happiness needs to be further examined. After World War II, the variables of happiness and life satisfaction were interchangeably utilized as indicators to investigate people’s subjective well-being empirically. Later, happiness and life satisfaction have been gradually identified as two distinctive variables to increase the reliability and validity empirically [107]. According to Ellison [8], life satisfaction, as an indicator of cognitive states, refers to implicit comparisons of life circumstances while happiness indicates the affective states responding to life quality spontaneously. Preliminary literature has shown that individual or cultural differences may lead to controversial judgments of subjective well-being when the variables of happiness and life satisfaction are applied respectively. For example, a higher evaluation of subjective well-being will be made by achievement-oriented individuals using the scale of life satisfaction [108] since life satisfaction is interpreted as outcomes of individual achievement. For this reason, sensation seekers may judge life satisfaction dissimilarly because they are more sensitive to affective indicators [109]. Recently, the internal interaction between life satisfaction and happiness has been debated and studied. Scholars, like Eldeleklioğlu [110], report that life satisfaction is predicted by subjective happiness significantly since cognitive evaluations are regarded as the outcome of affective states. Nevertheless, the literature also shows that need satisfaction works as a significant predictor of happiness in friendship quality [111,112]. The controversial results tend to be caused by different contexts; thus, it is necessary to reexamine the interaction between life satisfaction and happiness in the context of Buddhism-informed subjective well-being. 

Due to the spiritual power of adaptation and self-adjustment in Buddhism [10], it is anticipated that Buddhist religiosity tends to predict life satisfaction first, then influence happiness. Regarding this, the effect of the Buddhist symbols on happiness might be sequentially mediated by the perceived religiosity and then by life satisfaction. 

## 3. Hypotheses and Research Framework

According to the literature stated above, we suggest that the Buddhist symbols, such as the gesture symbol, could significantly raise people’s happiness, through the sequential mediating effects of life satisfaction and the perceived religiosity. Regarding this relationship, we developed the following hypotheses:

**H1:**  Aesthetic effect of Buddhist symbols has a positive impact on the perceived religiosity.

**H2:**  Aesthetic effect of Buddhist symbols has a positive impact on life satisfaction.

**H3:**  Aesthetic effect of Buddhist symbols has a positive impact on happiness.

**H4:**  Aesthetic effect of Buddhist symbols has an indirect and positive impact on happiness, mediated through the perceived religiosity.

**H5:**  Aesthetic effect of Buddhist symbols has an indirect and positive impact on happiness, mediated through life satisfaction.

**H6:**  Aesthetic effect of Buddhist symbols has an indirect and positive impact on happiness, sequentially mediated through, first, the perceived religiosity and then life satisfaction.

**Figure 1:**
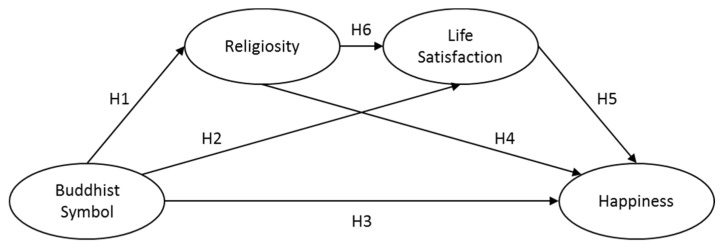
The theoretical framework of this study.

In order to respond to the research questions and test the hypotheses, an empirical study was conducted to explore the significant role of the Buddhist symbols in promoting religiosity and subjective well-being. Figure 1 depicts the theoretical framework with six hypotheses and the relationship between different variables. 

## 4. Method

### 4.1. Materials Selection as A Pilot Study

**Figure 2:**
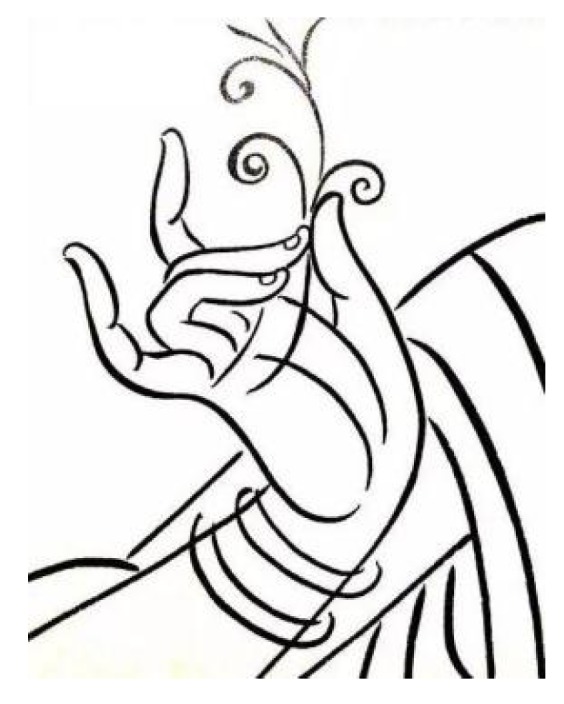
The gesture symbol shown on the mural painting at Dunhuang Cave No. 435.

**Figure 3:**
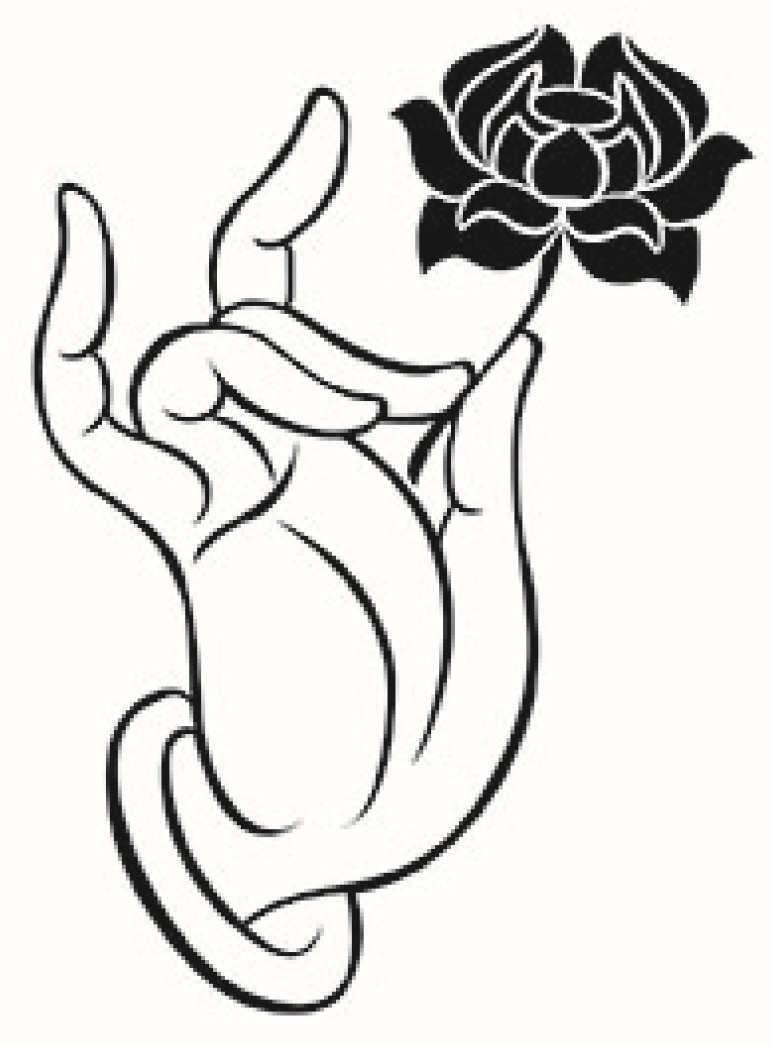
The adapted Buddhist symbol in our study.

In the statements above, the gesture symbols originating from Buddhist ceremonies are of importance in Buddhist artworks. Thus, we have adopted a gesture symbol of a hand holding a lotus flower (details are discussed in Section 2.1) in this study. This symbol is presented on the mural painting at Dunhuang Cave No.435 (Figure 2). To avoid visual distractions (for example, the robe pleats and imperfect lines) and ambiguities (for example, the flower in Buddha’s hand is uncharacteristic), one professional designer has adapted the symbol using professional software without damaging its figurative quality (see Figure 3). However, there still might be some co-founding factors that could potentially have an impact on people’s perceptions. For example, the impact of the elaborated symbol on people’s religiosity might not be consistent with the original symbol presented on the Dunhuang mural painting. Moreover, although the current study aims to explore the effect of Buddhist symbols on people’s subjective well-being, it is still unclear whether there might exist a significant improvement in perceived happiness compared with a neutral symbol [113]. Thus, a pilot study with a control group is needed to confirm that the Buddhist symbol could elicit higher happiness compared with the control group.

In order to control for these co-founding factors, we ran two pilot studies to explore whether the meaning of the adapted symbol is consistent with the original one and whether this symbol could elicit higher happiness compared with the control group. To specify, this pilot study contained two parts: we firstly ran an experiment to examine the difference in the perceptions of aesthetics and religiosity between two symbols with a follow-up interview, and then conducted another experiment to check the difference in happiness between Buddhist symbols and the neutral one. 

For pilot study I, 52 college students (mean age = 20.27; 46.2% male) were recruited in the experiment to confirm the consistent impact of the adapted symbol and the original one on people’s perceived religiosity and aesthetics. To be more specific, they were randomly assigned to two groups (each group contained 26 students) and then were asked to look at the given symbol and report their perceptions on religiosity and aesthetics. Results from a t-test confirmed differences for both perceived religiosity (t(50) = 0.783; *p* = 0.60) and aesthetics (t(50) = −1.117; *p* = 0.27) were both insignificant, suggesting the adapted symbol and original symbol shared a similar impact on perceived religiosity and aesthetics. Then, a follow-up interview was conducted to check the visual distractions and ambiguities of the two symbols. The questions included, for example, “Are there any visual distractions or ambiguities in this symbol? If so, what are they?”. Results from the interview further confirmed the robe pleats and other characteristics would have some visual distraction in the original symbol. Similarly, for pilot study II, 62 college students (mean age = 20.02; 66.1% female) were invited to see the Buddhist symbol or a neutral symbol (Wikimedia 2013; see the neutral stimuli in the Appendix A) and report their current feelings on happiness. Consistent with our predictions, the t-test showed a higher perception of happiness was associated with the Buddhist symbol (mean = 5.44), rather than the neutral symbol (mean = 4.51; *p* < 0.05).

### 4.2. Participants and Procedure in Main Study

In order to validate whether our survey was appropriately formed to test the hypotheses, 30 college students were recruited in the pre-study. Based on the feedback of the pre-study, we revised the survey questions to make sure that it was clearly and concisely stated as we predicted.

Given the theoretical framework in Figure 1, participants were invited through a survey service company (Wenjuanxing Survey Service) recruiting common people, rather than a student population, for generalizability purposes. Participants received a cash coupon as a reward for attending this research. In order to have a good representation of the population across China, we specifically asked the company to draw population equally from the northern and eastern parts of China, where the majority of Chinese people live. To specify, the northern part of China, in which Beijing is located, is considered the political center of China, while the eastern part of China, in which Shanghai is located, is considered the economic center of China [114]. In this way, respondents in these areas could represent the general Chinese people to a large extent.

To be more specific, participants in the main study were first approached to take part in this research and were then informed about the nature of this study. In addition, they were informed that they would be asked to examine this newly designed symbol for 15 s. Upon presentation of this symbol, they were asked to indicate the extent of their agreement to the questions in the survey. In total, the sample consisted of 220 unique responses. Of the participants, 129 (58.64%) were women, and 91 (41.36%) were men. The participants’ age ranged from 12 to 68: 62 were between the ages of 12 and 20 (28.18%); 96 were between the ages of 21 and 25 (43.64%), and 62 were between the ages of 26 and 68 (28.18%). Regarding residence places, 120 were from the eastern part of China (54.55%), 96 were from the northern part of China (43.64%), and 4 did not specify their places.

### 4.3. Measurement Items in Main Study

#### 4.3.1. Aesthetic Effect of the Buddhist Symbols

We measured the aesthetic effect of the Buddhist symbols using a 6-item Likert scale adapted from the related literature [115]. In this study, the visual aesthetics were emphasized (‘‘This Buddhist symbol looks attractive/ beautiful/ artistic/ pretty/ aesthetically appealing/ arousing’’, 1 = Strongly Disagree, 5 = Strongly Agree). Higher points in this construct suggest that the participants would have a higher level of visually aesthetic perception. 

#### 4.3.2. The Perceived Religiosity

The perceived religiosity was measured by a 5-item Likert scale adapted from previous work to be appropriate for the Buddhist context [116] (‘‘Buddhism is important to me’’/ “My ideas on Buddhism have a big influence on my view in other areas”/ “Were I to think about Buddhism differently, my whole life would be very different”/ “I often think about Buddhist matters”/ “Buddhism is one of the most important parts of my philosophy of life”, 1 = Strongly Disagree, 5= Strongly Agree). For instance, the original item “God is important to me” is adapted as ‘‘Buddhism is important to me’’. 

#### 4.3.3. Life Satisfaction

Life satisfaction was measured using the Satisfaction With Life Scale (SWLS) [117]. The SWLS has five items (‘‘The conditions of my life are excellent’’/ “In most ways my life is close to my ideal”/ “I am satisfied with my life”/ “So far, I have gotten the important things I want in life”/ “If I could live my life over, I would change almost nothing”, 1 = Strongly Disagree, 5= Strongly Agree). Higher points in this construct suggest that the participants would have a higher level of visually aesthetic perception. 

#### 4.3.4. Happiness

We measured happiness using a compact version of the Oxford Happiness Questionnaire (OHQ) [118]. It is a unidimensional scale with eight items (‘‘I have particularly happy memories of the past’’/ “I do not feel mentally alert”/ “I can fit in everything I want to”/ “I find beauty in some things”/ “I think I look attractive”/ “I am well satisfied with everything in my life”/ “I feel that life is very rewarding”/ “I feel particularly pleased with the way I am”, 1 = Strongly Disagree, 5= Strongly Agree). Higher points in this construct suggest that the participants would have a higher level of personal happiness. 

### 4.4. Design of the Study and Analysis of Data

This study used descriptive statistics, Pearson correlations, and sequential mediation analysis to examine the hypotheses. To specify, descriptive statistics aim to briefly describe the central tendency of the sample, which mainly contained the mean, the standard deviation, variance, and the kurtosis and skewness. Pearson correlations refer to a measure of the linear correlation between two variables. Factor analysis refers to a technique that could be conducted to reduce various factors into fewer ones. It could retrieve maximum common variance (MCV) from factors and forms a representative value. Cronbach’s alpha refers to a measurement of internal consistency of a particular construct, assessing the closeness of items in a group. Table 1 shows that all the constructs on this sample in this study have achieved satisfactory reliability (>0.80). Sequential mediation analysis works as an efficient tool in behavioral research aimed to explore the chain of reactions among different factors. All analyses were conducted using SPSS 22.0. No missing data was found in the current data sets. A total of 220 participants were included in the analyses. Before the main analysis, we checked the skewness and kurtosis of all the constructs [119]. Results showed the skewness ranged from –0.593 to 0.151, and the Kurtosis ranged from –0.278 to 1.40, which are all within the threshold, suggesting a normal univariate distribution that fulfills the assumption for further analysis [119,120]. 

Although we adopted the original scale from previous literature in this paper, we still needed to check all of the item loads of the specific construct via the factor analysis. According to the result of exploratory factor analysis, all the items of specific factors load a single construct. To specify, Aesthetics comprised 6 items that explained 76% of the variance with factor loadings from 0.843 to 0.901. Religiosity comprised 5 items that explained 69% of the variance with factor loadings from 0.762 to 0.891. Satisfaction with life comprised 5 items that explained 63% of the variance with factor loadings from 0.746 to 0.845. Happiness comprised 8 items that explained 54% of the variance with factor loadings from 0.644 to 0.818.

This study conducted analysis using the PROCESS macro for SPSS (Model 6; [121]). In order to analyze each indirect effect, PROCESS utilizes a bias-corrected confidence interval with bootstrapping (5000 resamples). If the confidence interval (CI) of the indirect effect does not contain 0, it shows that the mediation is significant. Compared with the Sobel test of mediation, bootstrapping is independent of the distribution assumptions of the indirect effect, thus achieving a superior statistical power [122]. In line with recent studies on sequential mediation approaches [123,124,125], we reported all the relationships in our framework: the total effect (c) and the direct effect (c’) in our model; the relationship between predictors and mediators (a1 and a2); the relationship between mediators and dependent variables (b1 and b2); the simple mediation relationship (a1–b1 and a2–b2); and the sequential mediation relationship (a1–d21–b2). According to the suggestions from Mancke et al. [125], the ratio of the indirect effect to the total effect is introduced when the total effect is more significant than the indirect effect with the same sign [122]. 

## 5. Results

Table 1 shows the means, standard deviations, Cronbach’s alpha, and correlations of different constructs. In total, 18.32% of the variance in happiness was explained in the current model. First, results showed that the aesthetic effects of the Buddhist symbol have a significant total effect on happiness (standardized effect = 0.323, *p* < 0.001). Then we tried to explore whether the perceived religiosity and life satisfaction would, uniquely and sequentially, mediate the effect of Buddhist symbols on happiness. Table 2 shows all the unique and sequential mediation analysis of our model. In line with our hypotheses, results showed a significant mediation (standardized effect = 0.179, *p* < 0.001), and the total mediation explained 55.4% of the total effect of the aesthetic effects of the Buddhist symbol on happiness. After controlling for the two mediators, results still showed a significant direct effect of the Buddhist symbol on happiness (standardized effect = 0.144, *p* < 0.001). To summarize, the two mediators in our model, perceived religiosity and life satisfaction, accounted for around half of the total effect of the Buddhist symbol on happiness.

Furthermore, we tried to analyze each indirect effect on the current model. Firstly, the perceived religiosity uniquely mediated the effect of a Buddhist symbol on happiness (independent of life satisfaction; standardized effect = 0.078). As the aesthetic effect of the Buddhist symbol increased, the perceived religiosity also increased (a1 effect = 0.345, *p* < 0.001). The perceived religiosity consecutively had a positive impact on happiness (b1 effect = 0.224, *p* < 0.001). Accordingly, the perceived religiosity played a unique mediating role in explaining the aesthetic effect of the Buddhist symbol on people’s happiness.

Similarly, life satisfaction also played a significant mediating role in the relationship between the Buddhist symbol and happiness independently of the perceived religiosity (standardized effect = 0.073). As the aesthetic effect of the Buddhist symbol increased, life satisfaction also increased (a2 effect = 0.215, *p* < 0.001). Life satisfaction, in turn, fed uniquely into happiness (b2 effect = 0.338, *p* < 0.001). Accordingly, life satisfaction played a simple mediating role in explaining the aesthetic effect of the Buddhist symbol on people’s happiness.

**Figure 4:**
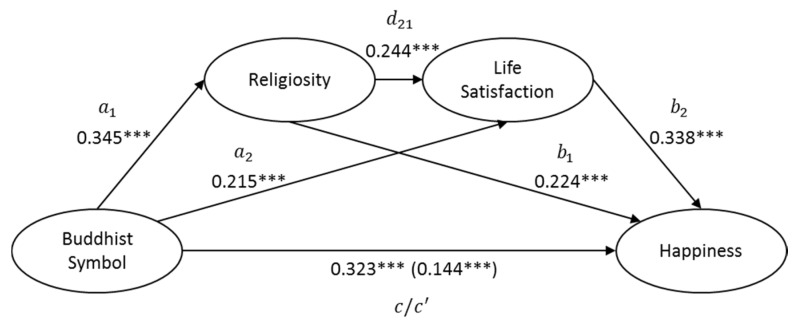
Mediating analysis of the links between the Buddhist symbol and happiness. **Note:** *** means significant p < 0.01

Lastly, we checked whether the perceived religiosity and life satisfaction could sequentially mediate the effect of Buddhist symbols on happiness. Results showed that the aesthetic effect of the Buddhist symbol significantly contributes to the perceived religiosity (a1 effect = 0.345, *p* < 0.001). In turn, the perceived religiosity contributed to life satisfaction (d21 effect = 0.244, *p* < 0.001), which finally predicted happiness (b2 effect = 0.338, *p* < 0.001). The sequential mediation was significant (standardized effect = 0.029). It confirmed that the aesthetic effect of the Buddhist symbol has an indirect and positive impact on happiness, sequentially mediated through the perceived religiosity and then life satisfaction. Figure 4. shows the theoretical framework of the current study.

## 6. Implications, Future Research, and Limitations

This study statistically revealed the significant influence of the Buddhist aesthetic symbol of a hand holding a lotus flower sequentially mediated by perceived religiosity and life satisfaction on perceived happiness. Thus, it makes several theoretical contributions in the area of religious symbols and their effect on subjective well-being. 

First, limited prior research has discussed religious symbols and their impact. To be more specific, although they might focus on the symbolism of specific religious symbols [52], they have largely neglected to discuss the influence of religious symbols on subjective well-being from the perspective of aesthetics. In particular, the aesthetic reaction towards a Buddhist symbol tends to play a crucial role in influencing people’s subjective well-being, not only for the believers, but also for ordinary people. In order to address this research gap, the current study attempts to contribute to the literature of religious symbols by showing the significant impact of aesthetic religious symbols on people’s happiness and identifying the sequential mediating role of religiosity and life satisfaction in this process.

Moreover, the result of this study is consistent with the recent findings in brain and cognition studies regarding the effect of aesthetics on human perception. For instance, based on fMRI data, Cupchik et al. [126] empirically showed that aesthetic perception and reaction to artwork is positively associated with the bilateral insula, attributing to the emotional experience. As a typical form of artwork [48,49,127], religious symbols could positively influence perceived religiosity and then have an impact on people’s subjective well-being in the context of Buddhism.

Also, by showing the sequential mediating role of religiosity and life satisfaction in this relationship, this study attempts to provide a relatively holistic picture of how a religious symbol affects people’s happiness, which is rarely discussed in the previous literature. Regarding this research gap, this paper has firstly validated the process that the Buddhist symbol of a hand holding a lotus flower could not only directly but also indirectly influence people’s subjective well-being by the mediating effect of religiosity. Lastly, regarding the ambiguous relationship between life satisfaction and happiness in the previous studies [110,111,112], the current study examined the subjective well-being as two different constructs, namely, life satisfaction, and happiness. It provided preliminary evidence that perceived religiosity and life satisfaction could work as significantly sequential mediators on happiness from a religious symbol perspective. 

The current study has some limitations, one being that since the participants recruited in the current study were mainly ordinary people in China rather than specifically Buddhists in China. Those people were called “spiritual but not religious” people, who are religious only to a limited extent. Although Buddhism has played an essential element in Chinese culture for centuries, it might also be important to investigate its impact on Buddhists specifically. In addition, all the variables seem to have an impact on happiness. However, we still do not know whether different factors shared the same importance or ultimately made a difference in influencing happiness. Accordingly, we would invite both common people and Buddhists for an interview study in the future. By conducting qualitative measures, we hope to obtain a more enriched picture and a deeper understanding of the effect and consequences of the use of Buddhist symbols on life satisfaction and happiness. In addition, the scales used in the current study are adapted from the related literature. Although it might help to maintain the content validity of the current empirical study, more direct questions regarding the perceptions of Buddhist symbols can reveal the specified impact of Buddhism. In addition, the current study might focus more on the short-term effect of religious symbols [128]. However, the long-term outcomes of religious symbols on happiness were rarely discussed in previous research. In future study, we could particularly adapt the items to address the research question more precisely and try to conduct a longitudinal study to examine whether continuous exposure to a religious symbol has an impact on the long-term evaluation of happiness and life satisfaction. Third, the duration of Buddhism-informed subjective well-being remains ambiguous in this study. Life satisfaction and happiness are long-term mental states. A specific investigation of how long the effects of Buddhist aesthetic symbols can continue to improve people’s psychological attitudes is needed in future study. Fourth, a comparison of responses from various Buddhist cultures (for example, Buddhists from Thailand, Tibetan, and Sri Lanka) and even non-Buddhists (for example, Christians and Muslims) will buttress the effects of Buddhist symbols and provide in-depth observations on the semiotic value. Future studies will scrutinize the effects of cultural differences on the perceptions of Buddhist symbols. Lastly, the current study considered the scenario with only one Buddhist symbol. However, complex or repetitious patterns are also often seen in Buddhist symbols. Although some prior research has explored the effect of complexity and repetition on people’s reactions [129], the influence within the context of Buddhism is still unclear. Thus, future study might use an experiment to explore further the effect of complexity and repetition on people’s religiosity and related subjective well-being.

## 7. Discussion

According to the results of the path analysis, all six hypotheses were statistically supported. Firstly, supporting H2 and H3, the Buddhist aesthetic symbol of a hand holding a lotus flower (Nianhua Shou in Chinese) tends to have a significant impact on life satisfaction and happiness. It suggests that the aesthetics of Buddhist symbols Mudra, such as a hand holding a lotus flower, plays a significant role in improving subjective well-being. This is consistent with the prior literature that a religious symbol could enhance people’s psychological enjoyment, pleasure [22,23,24,25,26,27,38,72], and experience [54]. 

Furthermore, supporting H1 and H4, the results have revealed that the Buddhist aesthetic symbol of a hand holding lotus flower could significantly enhance the perceived religiosity, and the perceived religiosity also has a significant influence on happiness. Similarly, supporting H5, the results show that life satisfaction is a significant mediator to predict happiness. 

Lastly, the empirical results have confirmed that the sequential mediating roles of the perceived religiosity and life satisfaction on happiness are also significant; thus, H6 was supported. As Buddhism advocates the spiritual power of adaptation and self-adjustment in life, which is closer to life satisfaction as a cognitive evaluation of life quality [10], the Buddhist aesthetic symbol of a hand holding a lotus flower could improve Buddhist religiosity first and then have an impact on perceptual life satisfaction. This result is consistent with the many prior studies that suggested a positive effect of perceived religiosity and life satisfaction on happiness [28,130]. 

## 8. Conclusions

This study provides preliminary evidence to confirm the theoretical relationship between Buddhist aesthetics and subjective well-being from the perspective of the religious symbol. Limited research has addressed how Buddhist aesthetics can shape religiosity and consequent perceptions of well-being. The current results statistically suggest the effectiveness of the Buddhist aesthetic symbol of a hand holding a lotus flower in enhancing perceived religiosity and happiness. It provides the first evidence of the mediating role of religiosity predicted by Buddhist aesthetic symbols in eliciting people’s happiness. This finding is consistent with previous research into the mediating role of religiosity on people’s subjective well-being [8,28]. The empirical analysis of the relationship between the Buddhist aesthetic symbol of a hand holding a lotus flower, religiosity, and happiness contributes to the theory of Buddhist aesthetics in the domain of religious symbols and subject well-being [89]. 

Previous research into the relationship between happiness and life satisfaction has revealed an ambiguous relationship. Some literature suggests that happiness works as a predictor for subjective satisfaction [110], while other researchers argue that life satisfaction functions as a significant factor influencing happiness [111,112]. Our result empirically supports the significant mediation role of life satisfaction in predicting happiness in the context of Buddhism. 

In addition, the present research further examined the inspiring role of religious symbols and the significant mediating roles of perceived religiosity and life satisfaction in the context of China, providing preliminary evidence that aesthetic religious symbols might have a potential impact on people’s religiosity, life satisfaction, and happiness in sequence.

## Figures and Tables

**Table 1:** Means, standard deviations, and correlations of different constructs.

Constructs	Range	Mean	SD	Cronbach’s Alpha	AE	RE	SA	HA
Aesthetics (AE)	1–5	3.36	0.99	0.94	–			
Religiosity (RE)	1–5	3.41	0.93	0.89	0.38***	–		
Satisfaction with Life (SA)	1–5	2.96	0.95	0.86	0.31***	0.32***	–	
Happiness (HA)	1–5	3.59	0.74	0.88	0.43***	0.49***	0.58***	–

Note: *N* = 220; * *p* < 0.1; ** *p* < 0.05; *** *p* < 0.01.

**Table 2:** The unique and sequential mediation analysis.

Mediation	Effect	SE	LLCI	ULCI	Ratio
Aesthetics -> Religiosity -> Happiness	0.08	0.03	0.03	0.14	0.24
Aesthetics -> Life Satisfaction -> Happiness	0.07	0.03	0.02	0.13	0.23
Aesthetics -> Religiosity -> Life Satisfaction -> Happiness	0.03	0.01	0.01	0.06	0.09
Total Indirect Effect	0.18	0.04	0.10	0.27	0.55

**Figure A1:**
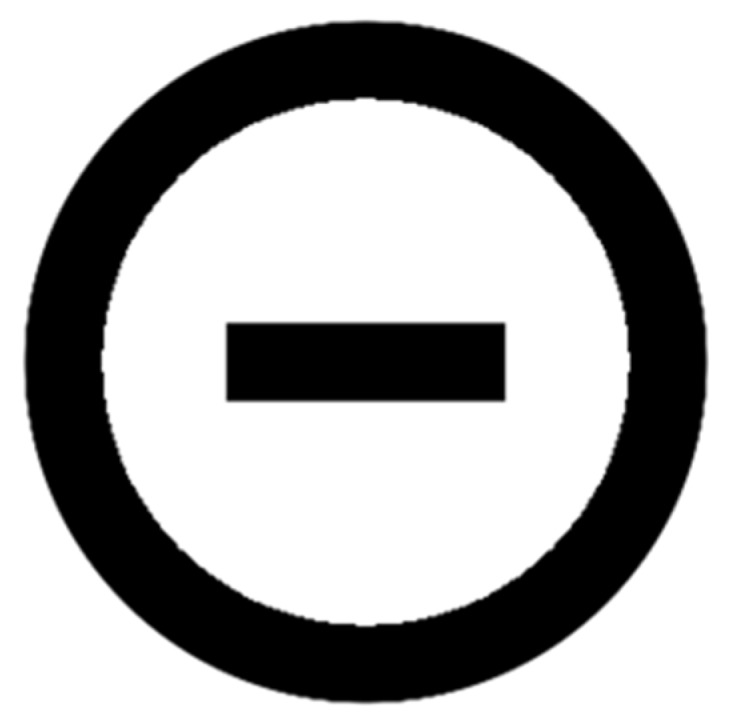
A neutral symbol used for the control group.
